# Auxin: small molecule, big impact

**DOI:** 10.1093/jxb/erx463

**Published:** 2018-01-02

**Authors:** Dolf Weijers, Jennifer Nemhauser, Zhenbiao Yang

**Affiliations:** 1 Wageningen University & Research, Laboratory of Biochemistry, The Netherlands; 2 Department of Biology, University of Washington, USA; 3 Institute of Integrative Genome Biology and Department of Botany and Plant Sciences, University of California, USA; 4 FAFU-UCR Joint Center for Horticultural Biology and Metabolomics Center, Haixia Institute of Science and Technology, Fujian Agriculture and Forestry University, China

**Keywords:** Auxin, AUXIN RESPONSE FACTOR (ARF), cell wall acidification, indole-3-acetic acid (IAA), lateral root formation, pin-formed1, PIN1, shoot meristem development

**Auxin research touches on a very wide variety of processes in plant development, and correspondingly a range of systems and approaches is revealing new understanding. This special issue spans this range, from research on mosses and liverworts which is revealing evolutionary aspects to the exquisite detail of signalling processes shown in Arabidopsis and agronomic potential in crops. As well as compiling and analyzing our current knowledge on auxin action, the reviews also provide a roadmap for future research.**

Since its discovery nearly a century ago (Thimann and Koepfli, 1935), auxin has risen to prominence as a plant signalling molecule, inspiring many to study its secrets. Past decades have seen a number of breakthroughs in the identification of a deceptively simple transcriptional response pathway (Weijers and Wagner, 2016), as well as cellular and molecular mechanisms of directional auxin transport (Adamowski and Friml, 2015), synthesis and inactivation (Korasick *et al.*, 2013). At the same time, auxin action has been reported in most, if not all, growth and developmental processes (Weijers and Wagner, 2016), in interactions with other hormonal signalling pathways (Vert and Chory, 2011), and even in the interaction with beneficial or pathogenic microorganisms and viruses (Boivin *et al.*, 2016). In fact, it is difficult to describe any plant process without some direct or indirect reference to auxin. The set of papers in this issue reflects this breadth of auxin research, and provides in-depth reviews in a wide range of aspects of auxin biology. While some of these represent ‘classical’ aspects of auxin action, others discuss recent progress in areas where the involvement of auxin has not yet been characterized in detail.

## Auxin maxima and cell growth

Arsuffi and Braybrook (2018) review research on one of the earliest, classical physiological responses to auxin: cell wall acidification leads to altered growth. This response has been studied for decades, initially using various physiological assays, which led to the formulation of a simple model. This model has seen many changes over the decades, mostly through genetic analysis of the auxin response. The authors bring together original ideas with recent exciting findings to propose a more complex scheme of cell wall acidification, and also highlight a number of outstanding questions.

Wang and Jiao (2018) discuss the role of auxin transport in shoot meristem development. One of the first mutants identified in the auxin field was *pin-formed1*, which produces naked, pin-like stems and no flowers. *PIN1* was later shown to encode an auxin transport protein, and mutations that reduced the biosynthesis of, or response to, auxin were found to cause similar defects. Thus, auxin is a potent regulator of flower formation at the shoot meristem. In recent years, genetic approaches, gene expression analysis and live imaging have led to new models as to how the local accumulation of auxin at the shoot meristem is controlled, and how these maxima trigger organ formation. Wang and Jiao provide an overview of this aspect of auxin action.

Similar to the shoot, local auxin accumulation in the root also has strong morphogenetic potential. Auxin maxima trigger several successive steps in the formation of lateral roots along the primary root. Separate responses provide context to the priming, initiation and emergence of lateral roots, and both the auxin response components and the downstream genes of these different steps have been characterized in recent years. Du and Scheres (2018) describe these latest findings and provide an integrated view of auxin-dependent lateral root formation.

Much auxin-related research has focused on the activity of the dominant naturally occurring auxin: Indole-3-Acetic Acid (IAA). Several synthetic analogues [including 2,4-dichlorophenoxyacetic acid (2,4-D) and 1-naphthaleneacetic acid (NAA)] have also been widely used. However, there are other naturally occurring molecules with auxin activity, and indole-3-butyric acid (IBA) has been the subject of active investigation for many years. IBA is nearly identical to IAA with the exception of an added CH_2_ group, yet the activity of IBA is rather different than IAA. Frick and Strader (2018) discuss the roles of IBA as a tightly regulated auxin storage form that allows spatio-temporal control of auxin levels during plant development, particularly in the elaboration of the root system. A key question they discuss is whether IBA action fully depends on enzymatic conversion to IAA. In addition, the authors suggest that the liberation of active auxin from IBA can be strongly modulated by environmental stresses.

## The nuclear auxin response

Auxin is well known for its ability to regulate numerous growth and developmental processes. Many such outputs depend on the modification of gene expression programs. Auxin-dependent AUXIN RESPONSE FACTOR (ARF) transcription factors are the final step that selects which genes are auxin-regulated and how. Roosjen *et al.* (2018) provide an extensive overview of the ARF transcription factor family, their specificity of DNA binding and modes of action. The authors propose that ARFs possess an intrinsically disordered domain and speculate on how this might mediate gene regulation.

The ARFs have surfaced as the key auxin-dependent transcription factors, and a binding site for these proteins has been defined. However, many auxin-regulated genes do not display clear ARF target sites. Cherenkov *et al.* (2018) describe a bioinformatic meta-analysis of a large number of auxin-related transcriptomics datasets to identify hexamer sequences that are enriched in auxin-dependent genes. In addition to the well-known ARF binding site, the authors have found several other motifs that are associated with auxin responses. These motifs can be correlated to chromatin properties, as well as to potential binding of other transcription factors. This offers a framework to consider new regulators of auxin-dependent genes.

## Interactions with other hormones, other organisms and the environment

Through its central position in the regulation of growth and development, the auxin response is modulated by many other signalling pathways. Han and Hwang (2018) provide an overview of the intersections and interactions of the auxin response pathway with other hormonal signalling components. Mroue *et al.* (2018) review the way in which environmental triggers modulate auxin homeostasis, and focus on the modulation of auxin biosynthesis.

Clearly, there is widespread regulation of auxin homeostasis by a variety of environmental stimuli, thus translating external conditions to coordinated changes in growth and development. One such environmental condition is light quality. The ratio of red to far-red light is sharply decreased when plants are shaded by neighbours. Plants detect these differences in light quality and use this as a signal to enhance stem growth. Iglesias *et al.* (2018) describe how light quality directs altered growth through changing auxin biosynthesis and transport. A central module that connects light receptors to a light-regulated transcription factor and to auxin biosynthesis genes plays a central role in this response and, at the same time, light-regulated auxin transport changes help create local auxin maxima for directional growth.

Biotic environmental factors also modulate plant growth and development through auxin activity. A good example is the formation of root nodules by legumes in symbiosis with *Rhizobium* bacteria. Kohlen *et al.* (2018) describe how the interaction between root cells and bacterium-derived and bacterium-induced signals involves changes in auxin transport and response to locally activate cell divisions and generate a nodule. Finally, the interaction with bacterial plant pathogens also involves auxin activity. A famous example is the crown gall-inducing *Agrobacterium tumefaciens*, which transfers hormone biosynthesis genes, including auxin biosynthesis genes, to the plant genome and through this triggers cell division. However, there are several other cases of bacteria manipulating auxin action, for example by modulating the auxin response to alter growth and development or to subvert defence responses. Kunkel and Harper (2018) discuss the ways in which bacterial pathogens manipulate auxin biology to facilitate their survival and viability in plants.

## Evolution and divergence of auxin biology

Most auxin research has been performed in the dicot model species *Arabidopsis thaliana*, although earlier, physiological research was done in a range of species. Arabidopsis is not of agronomic importance, but clearly the significant impact of auxin action throughout plant life means that agronomically relevant traits in crops are also influenced by auxin. Wang *et al.* (2018) review what rice research has contributed to our understanding of auxin biology and, more importantly, discuss which quality and yield traits in rice are controlled by auxin. This paves the way for targeted crop modification based on changing auxin activity.

Auxins have been used in agriculture and horticulture for decades, both to control plant growth and notably also to kill weeds. Auxin-based herbicides are plentiful, but the chemical basis for herbicide action is not always obvious. Quareshy *et al.* (2018) have compiled chemical and physical information on all major auxinic herbicides and describe a chemi-informatic analysis of these properties in relation to the herbicidal activity of the compounds. This rich source of information will help further develop agrochemistry based on auxin action.

Given the broad range of activities in flowering plants, a central question in auxin biology is how this system emerged and evolved complexity. Two reviews discuss auxin activity and response in basal land plants. Thelander *et al.* (2018) review how auxin controls development in the moss *Physcomitrella patens*. Essentially all growth and developmental programs depend in one way or another on auxin biosynthesis, transport and/or responses. Interestingly, the auxin response system in this moss is considerably simpler than those in flowering plants, and hence this model system should facilitate understanding of how diversity in auxin responses is generated. Kato *et al.* (2018) mostly focus on the liverwort *Marchantia polymorpha*, which probably diverged before the mosses and has an even simpler auxin response system, and discuss the conservation of mechanisms in auxin responses.

## New tools

Drugs have been instrumental in dissecting mechanisms of auxin action. Compounds that inhibit auxin transport, for example, have helped explain how it is transported and how this controls growth and development. Curiously though, the molecular mechanism through which such compounds act is not always clear, even if they are widely used. Teale and Palme (2018) review the current literature on the working mechanisms of 1-N-naphthylphthalamic acid (NPA), a widely used auxin transport inhibitor.

In addition to the ‘classical’ compounds used in auxin biology, there have been several endeavours to screen for novel small molecules that modify auxin transport, biosynthesis or responses. Clearly, well-characterized compounds will be invaluable tools in generating an even deeper understanding of auxin action, much as auxin transport inhibitors have done in the past. Ma *et al.* (2018) discuss the chemical biology and chemical genetics approaches that have been taken to dissect auxin action.

## Outlook

The papers in this special issue compile and analyze our current knowledge of auxin action. They also provide a roadmap for future research. Clearly, the mechanistic understanding of auxin biosynthesis, breakdown and response has been developed to nearly atomic resolution through the availability of crystal structures of the main proteins involved (Parcy *et al.*, 2016). Similar molecular – and thus mechanistic – detail is lacking for auxin transport, and it is evident that such information would help us understand not only the mechanisms of auxin transport, but also its regulation by various endogenous and exogenous signals. Perhaps such structural information would also help rationalize the effects of well-established drugs and small chemical compounds on auxin transport.

After a strong focus on generic principles in auxin biology, recent years have brought an appreciation of the diversity of hormone action, in terms of both the multiple growth and developmental responses in model plants and the evolutionary context of auxin action. Now that genomic technologies allow the analysis of the local auxin response (Bargmann *et al.*, 2013; Möller *et al.*, 2017), it is likely that the molecular basis for the diversity of auxin-triggered events during development will become clearer. At the same time, genomic technologies are allowing the extraction of detailed information from a much wider range of species than just the genetically tractable models. This allows the first glimpses into cross-species diversity and evolution of auxin action, but it is expected that coming years will bring deep insight into the origin and evolutionary history of auxin action as well as helping us to understand species-specific aspects of auxin biology. These, in turn, may help this research field to turn a rich history of discovery into approaches to improve crops for the future.
